# 1439. Latent tuberculosis infection treatment location and association with care completion

**DOI:** 10.1093/ofid/ofac492.1268

**Published:** 2022-12-15

**Authors:** Jeffrey Campbell, Mary Tabatneck, Grete Wilt, Grete Wilt, Mingwei Sun, Wei He, Nicholas Musinguzi, Bethany Hedt-Gauthier, Gabriella S Lamb, Donald Goldmann, Vishakha Sabharwal, Thomas J Sandora, Jessica Haberer

**Affiliations:** Boston University School of Medicine, Boston, Massachusetts; Boston Children’s Hospital, Boston, Massachusetts; Harvard School of Public Health, Boston, Massachusetts; Harvard School of Public Health, Boston, Massachusetts; Boston Children's Hospital, Boston, Massachusetts; Massachusetts General Hospital, Boston, Massachusetts; Mbarara Univeristy of Science and Technology, Mbarara, Mbarara, Uganda; Harvard Medical School, Boston, Massachusetts; Boston Children's Hospital, Boston, Massachusetts; Harvard Medical S, lexington, Massachusetts; Boston University, Boston, Massachusetts; Boston Children's Hospital, Boston, Massachusetts; Massachusetts General Hospital, Boston, Massachusetts

## Abstract

**Background:**

Location and type of clinic where pediatric latent TB infection (LTBI) care is provided are associated with treatment completion and retention in care. Prior research has not evaluated joint clinical management occurring between care settings. Understanding care transfer dynamics and accessibility of clinics can inform pediatric LTBI care service delivery.

**Methods:**

We conducted a retrospective cohort study of LTBI in children 0-17 years old who were prescribed outpatient treatment in two Boston-area health systems from 2017-2019. We defined “initial clinical setting” (categorized as primary care or TB/infectious diseases clinic) as the location where the first LTBI medication was prescribed. Through chart review, we determined if care was transferred to a different (“final”) clinic setting during treatment. We calculated driving time between a child’s home address and initial and final treatment clinics. The primary outcome was frequency of care transfer after starting treatment. In a secondary analysis, we used two multivariable logistic regression models (adjusted for age, sex, and use of rifamycin-based treatment) to evaluate associations between completion and distance to and type of initial and final treatment clinic.

**Results:**

We identified 142 children who started LTBI treatment as outpatients; 110 started treatment in primary care clinics and 32 in TB/infectious diseases clinics. Overall, 20/142 (14%) transferred TB care to a different clinic after starting treatment. A total of 101/142 (71%) patients completed treatment. Neither initial treatment location nor driving time to initial clinic were significantly associated with treatment completion (**Table 1**). However, final treatment in a TB clinic was associated with higher odds of treatment completion than final treatment in a primary care clinic (aOR 2.71 [95%CI 1.06-6.91], P=0.04); time to clinic was not associated with completion (**Table 2**).

**Figure 1.**
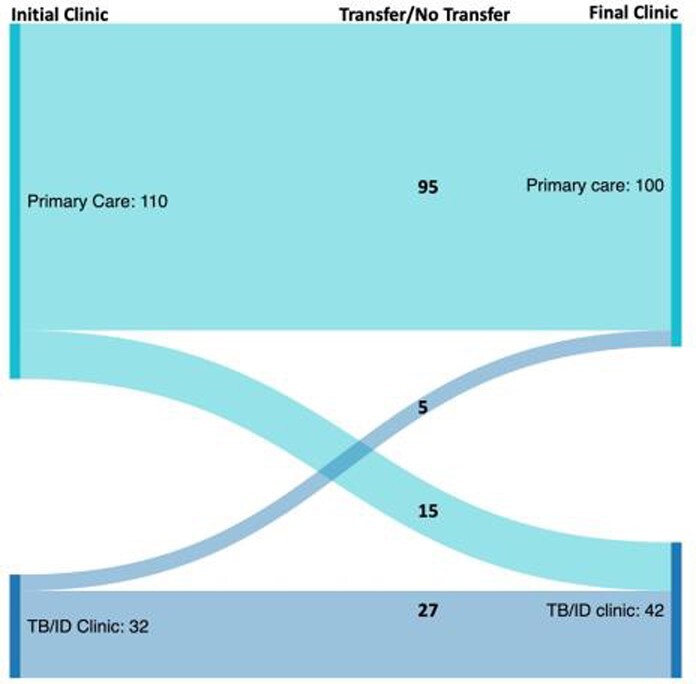
Patient transfers after starting LTBI treatment.

**Table 1.**
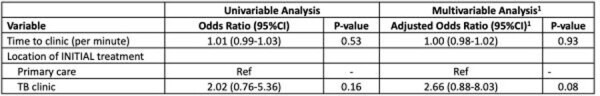
Initial treatment location: Univariable and multivariable analysis of factors associated with treatment completion. 1Adjusted for time to clinic and location of initial treatment as well as age, sex, and use of rifamycin-based treatment.

**Table 2.**
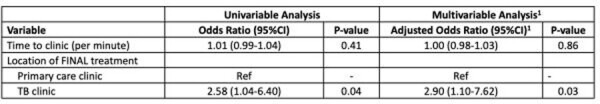
Final treatment location: Univariable and multivariable analysis of factors associated with treatment completion. 1Adjusted for time to clinic and location of final treatment as well as age, sex, and use of rifamycin-based treatment.

**Conclusion:**

Among children with LTBI in a large metropolitan area, more patients received treatment in primary care clinics than in TB clinics. Care transfers were relatively uncommon after starting treatment. A TB clinic as a final treatment location was associated with increased odds of treatment completion.

**Disclosures:**

**Jessica Haberer, MD, MS**, Merck: Advisor/Consultant|Natera: Stocks/Bonds.

